# Halitosis, Oral Health-Related Quality of Life, and Active Dental Treatment: A Prospective Observational Comparative Study Across Periodontal, Prosthodontic, and Orthodontic Modalities

**DOI:** 10.3390/healthcare14121643

**Published:** 2026-06-10

**Authors:** Romina Georgiana Bita, Otilia Cornelia Boloș, Edida Maghet, Adrian Boloș, Raluca Briceag, Bogdan Andrei Bumbu

**Affiliations:** 1 2nd Department, Radiology and Medical Imaging, General and Dento-Maxillary Imaging, Dental Medicine Faculty, “Victor Babeș” University of Medicine and Pharmacy, 300041 Timișoara, Romania; romina.bita@umft.ro; 2 Department of Dental Aesthetics, Faculty of Dental Medicine, “Victor Babeș” University of Medicine and Pharmacy, 300041 Timișoara, Romania; bolos.otilia@umft.ro; 3 Faculty of Dental Medicine, “Victor Babeș” University of Medicine and Pharmacy, 300041 Timișoara, Romania; edida.maghet@umft.ro; 4 Department of Oral Rehabilitation, Faculty of Dental Medicine, Specialization of Dental Technology, “Victor Babeș” University of Medicine and Pharmacy, 300041 Timișoara, Romania; 5 Faculty of Dental Medicine, Ovidius University of Constanța, 7 Ilarie Voronca Street, 900684 Constanța, Romania; 6 Department of Dental Medicine, Faculty of Medicine and Pharmacy, University of Oradea, 410073 Oradea, Romania; bogdanbumbu@uoradea.ro

**Keywords:** halitosis, quality of life, periodontal debridement, orthodontic appliances, fixed, dental prosthesis

## Abstract

Background and Objectives: Halitosis is a prevalent oral concern that meaningfully affects oral health-related quality of life (OHRQoL), yet how active dental treatment is associated with short-term changes in the objective–subjective halitosis–QoL nexus remains poorly quantified. Interpretation is complicated by the multifactorial nature of malodor and by baseline differences between patients selected for different dental procedures. We compared changes in volatile sulfur compound (VSC) emissions, organoleptic ratings, tongue-coating burden, and OHIP-14 across three contrasting treatment modalities and explored whether VSC change statistically accounted for OHRQoL change. Methods: In a non-randomized prospective comparative study, 119 adults (18–67 y) commencing one of three procedures were assessed at baseline and at 8 weeks: scaling and root planing (Group A, n = 42), fixed prosthodontic rehabilitation (Group B, n = 38), or fixed orthodontic appliance bonding (Group C, n = 39). Outcomes included Halimeter^®^ VSC (ppb), Rosenberg organoleptic score (0–5), Winkel tongue-coating index (TCI), self-perceived halitosis, and OHIP-14 total and seven-domain scores. Mixed-design ANOVA, ANCOVA, prespecified multivariable regression, mediation (5000 bootstrap resamples), receiver operating characteristic analysis, and four-class latent class analysis were performed. A sensitivity-analysis framework including expanded covariate adjustment, propensity-score overlap weighting, and baseline-severity strata was also applied to address residual baseline imbalance. Secondary mediation, ROC, and latent-class analyses were considered exploratory. Results: At 8 weeks, VSCs fell by 116.4 ± 38.7 ppb in Group A and 35.4 ± 29.1 ppb in Group B but rose by 34.3 ± 28.6 ppb in Group C (*p* < 0.001). OHIP-14 improved by 10.3 and 4.9 points in A and B and worsened by 3.7 in C (*p* < 0.001). ΔVSC correlated with ΔOHIP-14 (ρ = 0.51, *p* < 0.001) and most strongly with the psychological discomfort domain (ρ = 0.58). VSC change mediated 35.1% of the periodontal-versus-orthodontic association on QoL (indirect β = −4.7; 95% CI −6.3 to −3.1). Because VSC and OHIP-14 changes were measured over the same interval, mediation was interpreted cautiously. A ΔVSC threshold of −63 ppb predicted clinically meaningful OHIP-14 improvement (AUC = 0.81). Latent class analysis identified four distinct responder phenotypes. The cutoff and responder classes were internally derived and require external validation. Sensitivity analyses preserved the direction of the primary contrasts, but residual confounding remains possible. Conclusions: Treatment modality was associated with the direction and magnitude of halitosis and QoL change, with orthodontic patients constituting a vulnerable subgroup. Targeted oral-hygiene reinforcement during fixed-appliance therapy is warranted.

## 1. Introduction

Halitosis is a multifactorial condition with intra-oral origins accounting for approximately 80–90% of cases, predominantly through bacterial degradation of sulfur-containing substrates on the dorsum of the tongue and within periodontal niches [1]. Population prevalence estimates span 22–50%, varying by detection method and demographic context, including how halitosis is defined and graded and whether transient morning malodor is separated from persistent clinical halitosis [2], and the resulting psychosocial burden frequently extends beyond the strictly clinical domain into avoidance behaviors, dating apprehension, and occupational self-consciousness [3]. Although mechanistic pathways linking sulfur-gas chemistry to subjective complaint have been mapped in cross-sectional cohorts, far less is known about how active dental treatment—as opposed to ambient hygiene practices—reshapes both biochemical odor and patient-reported wellbeing in the weeks immediately following intervention; moreover, the longevity of any improvement remains uncertain when malodor has multiple simultaneous contributors, including periodontal inflammation, tongue coating, salivary flow, diet, smoking exposure, prosthetic surfaces, orthodontic appliances, and patient perception.

The classic dental-care literature has approached halitosis primarily through the lens of periodontal disease, where elevated probing depths, supragingival plaque, and bleeding on probing are consistently associated with two- to three-fold elevations in volatile sulfur compound (VSC) concentrations [4]. Mechanical debridement, scaling and root planing, and chlorhexidine adjuncts have all been shown to reduce VSC output and organoleptic scores, often within a single clinical cycle [5,6]. Adjunctive tongue-cleaning measures further enhance these gains, although effect estimates remain heterogeneous across pooled analyses [7]. What remains underexplored is whether these objective gains translate proportionately into perceptible improvements in oral health-related quality of life (OHRQoL), the construct most relevant to the patient’s lived experience and adherence to long-term care.

In contrast, prosthodontic rehabilitation occupies an intermediate position. Fixed prostheses—single crowns, bridgework, implant-supported restorations—correct mastication, esthetics, and occlusal vertical dimension yet introduce new plaque-retentive surfaces (margins, embrasures, pontic undersurfaces) that can paradoxically impede oral clearance [8]. The net effect on halitosis is therefore equivocal in the published literature, with small case series reporting either modest VSC reduction (attributable to caries elimination and improved flossability) or unchanged sulfur-gas profiles (attributable to retention sites) [9]. The QoL trajectory after prosthodontic treatment, conversely, is generally favorable due to functional and esthetic gains, raising the possibility of a subjective improvement that outpaces the underlying chemical change.

The third modality—fixed orthodontic appliances—presents the inverse problem. Brackets, archwires, ligatures, and elastomeric modules collectively expand plaque-retentive surface area by an estimated 1.5- to 2.0-fold and impair the access of conventional oral-hygiene tools [10]. Within four to twelve weeks, increases in *Streptococcus mutans* counts, *Prevotella* spp. proliferation, and gingival inflammation are well documented [11]. Oral malodor is also linked to anaerobic and proteolytic taxa such as *Porphyromonas gingivalis*, *Treponema denticola*, *Fusobacterium nucleatum*, *Prevotella intermedia*, and *Solobacterium moorei*, which can generate hydrogen sulfide, methyl mercaptan, dimethyl sulfide, and related odorants from sulfur-containing substrates. Whether these microbiological shifts translate into a clinically meaningful rise in VSCs and a worsening of OHRQoL has been suggested anecdotally but not rigorously quantified using standardized halimetry plus a validated QoL instrument in the same cohort [12].

A further unanswered question concerns the concordance between objective halitosis and subjective experience. Across cross-sectional samples, correlations between Halimeter readings and self-perceived halitosis hover between 0.30 and 0.55, indicating substantial discordance [13]. Some patients carry a heavy chemical load yet remain unbothered; others perceive offensive breath despite low VSCs. This decoupling has clinical and ethical implications—the former group is at risk of social harm without subjective alarm, the latter at risk of overtreatment, halitophobia, and dependency on cosmetic products [14]. No published study has, to our knowledge, examined whether the direction of change after dental treatment is similarly discordant or whether different procedures cluster patients into distinguishable response phenotypes.

Addressing these gaps, the present prospective observational study compared 119 patients undergoing periodontal, prosthodontic, or orthodontic treatment at baseline and at eight weeks. The 8-week endpoint was chosen to provide a common early follow-up interval across all three modalities: initial periodontal healing and inflammation reduction are expected by this stage, prosthodontic patients have completed early adaptation to fixed restorations, and orthodontic patients are within the plaque-retentive adaptation period after appliance bonding. This interval was not intended to replace the 3-month periodontal maintenance assessment or to characterize long-term orthodontic adaptation. We hypothesized that (i) the three modalities would produce divergent changes in objective and subjective halitosis indicators; (ii) ΔVSC would partially mediate procedure-related changes in OHIP-14; (iii) thresholds of VSC reduction would predict the minimal clinically important difference (MCID) for OHIP-14; and (iv) latent class analysis would identify clinically meaningful responder phenotypes that cut across treatment groups. Because treatment was clinically indicated rather than randomized, all procedure-related findings were interpreted as associations. Confirming these hypotheses would provide actionable guidance for procedure-specific halitosis counseling and post-operative monitoring.

## 2. Materials and Methods

### 2.1. Study Design and Ethical Considerations

This was a prospective, observational, three-arm comparative study conducted between February 2024 and March 2026 in the Faculties of Dental Medicine of Timișoara and Oradea, Romania. The protocol adhered to the Declaration of Helsinki and was approved by the Ethics Committee for Scientific Research of the “Victor Babeș” University of Medicine and Pharmacy Timișoara, Romania, before recruitment (Approval No. 1284/11 January 2024). The reporting conforms to the STROBE checklist for observational studies [15]. All participants received oral and written explanation of the study procedures, time commitment, voluntary nature of participation, and right to withdraw without compromise to clinical care and provided written informed consent before any data were recorded.

The PICO framework was as follows: the population comprised adult dental patients (18–67 years) commencing one of three index treatments. The exposures of interest were (i) periodontal therapy delivered as full-mouth scaling and root planing across two appointments, (ii) fixed prosthodontic rehabilitation involving cementation of a definitive metal-ceramic or zirconia crown/bridge, or (iii) initial bonding of fixed orthodontic appliances on the maxillary and mandibular arches. The comparators were the other two arms of the cohort. The outcomes were dual: objective—total VSC concentration in mouth-air, organoleptic score, tongue-coating index—and subjective—OHIP-14 total and domain scores, single-item self-perceived halitosis. The time points were baseline (T0, immediately before the indicated treatment) and 8 weeks post-treatment (T1). De-identified data were stored on an encrypted institutional server with role-based access and were retained for ten years per local regulation.

### 2.2. Participants, Inclusion, and Exclusion Criteria

Eligible adults were aged 18–67 years, possessed a minimum of 20 natural teeth, were medically stable (American Society of Anesthesiologists class I–II), and were scheduled to commence one of the three index procedures within two weeks of recruitment. Patients in the periodontal arm were required to present with generalized stage II periodontitis (CDC/AAP criteria) and to undergo full-mouth scaling and root planing in two appointments separated by 24–48 h. Prosthodontic arm patients required at least one fixed restoration (crown, fixed partial denture, or implant-supported single crown) to be delivered in the upper anterior or premolar zone. Orthodontic arm patients were fitted with conventional pre-adjusted edgewise appliances on both arches and had not previously worn fixed appliances.

Exclusion criteria were systemic disease known to affect saliva (Sjögren syndrome, uncontrolled diabetes, head-and-neck radiotherapy); chronic medication with documented xerogenic effect (anticholinergics, certain antidepressants, antipsychotics); antibiotic use within four weeks; current pregnancy or lactation; psychiatric diagnosis of halitophobia (ICD-11 6B23); active oropharyngeal infection; smoking >10 cigarettes per day because heavier tobacco exposure can independently elevate oral malodor and confound VSC measurement; complete denture wear; and inability to attend the 8-week follow-up. Patients smoking fewer than 10 cigarettes per day were not excluded in order to preserve external validity, but smoking status was retained as a covariate. Urinary cotinine was not measured; therefore, smoking exposure was based on self-report and is acknowledged as a potential source of exposure misclassification. Of 142 individuals screened, 23 were excluded (15 not meeting criteria, 6 declined, 2 unable to commit to follow-up). The 119 enrolled participants completed both assessment time points; no attrition occurred between T0 and T1.

### 2.3. Examination Protocol and Variables

All assessments were conducted in a dedicated, climate-controlled (21–23 °C, 40–55% relative humidity), low-odor examination room between 08:00 and 11:00 to minimize circadian variation. Participants were instructed to refrain from eating, drinking (other than water), oral-hygiene activities, chewing gum, smoking, vigorous physical exercise, and use of scented personal-care products from midnight prior to each visit; adherence was confirmed by a brief questionnaire on arrival and any non-adherence prompted rescheduling within 72 h. At treatment initiation, all participants received standardized written and verbal hygiene instructions adapted to their modality, including twice-daily brushing, interdental cleaning, tongue cleaning, avoidance of unsupervised antiseptic mouthwash changes, and specific instructions for appliance or prosthesis cleaning. At the 8-week visit, participants reported adherence, tongue-cleaner use, mouthwash use, appliance/prosthesis cleaning, and recent diet using the same brief checklist. Two examiners, calibrated against a senior reference examiner across 12 pilot subjects (intra-class correlation ≥ 0.85 for all continuous indices, Cohen’s κ ≥ 0.78 for binary items), conducted all measurements blinded to the participant’s treatment arm where the device permitted; however, complete examiner masking was not feasible during oral examination because orthodontic appliances and prosthetic restorations could be visible. To reduce observer bias, organoleptic ratings and TCI were scored independently by both examiners, and disagreement was resolved by consensus before database entry.

The order of measurement was standardized: (i) self-administered OHIP-14 questionnaire on a tablet device, scored according to the original short-form instrument [16,17,18]; (ii) self-perceived halitosis single item (“Do you believe your breath usually smells unpleasant to others?”, yes/no); (iii) two-minute mouth-closed rest; (iv) total VSC measurement using a portable Halimeter^®^ AC1000 (Interscan Corp., Los Angeles, CA, USA), three consecutive readings 60 s apart, mean recorded in ppb, following the validated protocol of Rosenberg and colleagues [16]; the Halimeter^®^ was selected because it is feasible for repeated chairside measurements, but it reports total sulfur signal and cannot speciate hydrogen sulfide, methyl mercaptan, and dimethyl sulfide as gas chromatography can; (v) organoleptic rating on Rosenberg’s 0–5 scale, each examiner scoring independently and consensus reached for any disagreement (weighted κ = 0.84); (vi) Winkel tongue-coating index (TCI) computed as the mean of six dorsal-zone scores [17]; (vii) plaque index (Silness–Löe) and bleeding-on-probing percentage; (viii) collection of unstimulated saliva by 5-min passive drool. For clinical interpretation of measurement error, the mean absolute interexaminer differences during calibration were 8.6 ± 6.2 ppb for VSC readings, 0.28 ± 0.31 points for organoleptic score, and 0.11 ± 0.09 points for TCI. Covariates recorded at baseline included age, sex, body-mass index, smoking status, self-reported nocturnal mouth breathing, education years, and previous halitosis-targeted product use.

### 2.4. Statistical Analysis

Sample size was estimated a priori using G*Power 3.1.9.7 for a one-way ANOVA with three groups, assumed effect size f = 0.32, α = 0.05, and power 1 − β = 0.85, which yielded a minimum of 105 participants [19]. We enrolled 119 to accommodate up to 12% attrition. This calculation was intended for the primary between-group comparison of VSC and OHIP-14 change. Mediation, ROC, and latent-class analyses were treated as secondary exploratory analyses and were not independently powered as confirmatory endpoints; their adequacy was therefore judged by confidence intervals, bootstrap precision, entropy, and consistency with clinical plausibility rather than by formal hypothesis-confirmatory power. Accordingly, *p* values from these secondary analyses were reported descriptively and were not used to claim independent confirmatory evidence. Analyses were conducted in IBM SPSS Statistics v29.0 (Armonk, NY, USA) and R 4.3.2 (R Core Team, Vienna, Austria) using packages psych, lavaan (mediation), pROC (ROC), and poLCA (latent class). Normality was checked with Shapiro–Wilk and visual inspection of Q-Q plots; homogeneity of variance with Levene’s test; multicollinearity in regression models with variance inflation factor (VIF) < 2.5. VSC and OHIP-14 totals were normally distributed within each cell after log-back-checks; raw values were therefore retained for primary analyses while sensitivity analyses on log10-transformed variables produced concordant inferences.

Continuous data are presented as mean ± SD and categorical data as frequency (%). Between-group comparisons used one-way ANOVA with Tukey HSD post hoc tests when assumptions held, Welch’s ANOVA with Games–Howell when variance differed, and Kruskal–Wallis with Dunn’s adjustment for skewed indices. Categorical comparisons used Pearson χ^2^ or Fisher’s exact test as appropriate. Within-group T0–T1 changes used paired-samples t-tests with Cohen’s d_z effect sizes. Group × time interactions were evaluated by mixed-design ANOVA. ANCOVA adjusted T1 outcomes for baseline values. Spearman’s ρ quantified bivariate associations. Multivariable linear regression predicted ΔOHIP-14 from procedure group, ΔVSC, baseline OHIP-14, and demographic covariates using a prespecified clinically informed model retaining procedure group, ΔVSC, baseline OHIP-14, age, sex, BMI, and mouth breathing regardless of statistical selection. Mediation was tested using the bias-corrected percentile bootstrap (5000 resamples) implemented in the lavaan package, complemented by the Sobel z-test. Receiver operating characteristic analysis derived the optimal ΔVSC cutoff for predicting MCID OHIP-14 improvement (≥5-point reduction) by Youden’s index, with AUC pairs compared via DeLong’s test. Latent class analysis enumerated 1- to 5-class solutions on standardized ΔVSC and ΔOHIP-14, selecting the model with lowest Bayesian Information Criterion, entropy ≥ 0.80, and significant Lo–Mendell–Rubin likelihood ratio test. All tests were two-sided with α = 0.05; Bonferroni correction was applied within multiple-comparison families. Because treatment allocation was not randomized and baseline imbalance was expected, effect estimates were interpreted as adjusted associations. To address the reviewer-noted baseline imbalance, we added three sensitivity analyses: (i) an expanded covariate model additionally adjusting for BMI, education, plaque index, bleeding on probing, mouth breathing, and baseline OHIP-14/VSC as appropriate for the outcome; (ii) a propensity-score overlap-weighted model using age, sex, BMI, education, smoking status, plaque index, bleeding on probing, mouth breathing, and baseline outcome value to emphasize the portion of the sample with greatest covariate overlap; and (iii) baseline-severity strata for VSC and OHIP-14 to examine whether the direction of change persisted among patients with comparable starting burden. Conventional 1:1 propensity-score matching was not used as the primary adjustment because the clinically indicated treatment groups showed limited demographic and periodontal overlap and matching would have discarded a substantial portion of this modest cohort. These sensitivity analyses were performed as robustness checks and were not interpreted as eliminating residual confounding. No participant was lost to follow-up and no primary-outcome item-level missingness was identified, so imputation was not required.

## 3. Results

The three procedure groups differed substantially in age and BMI, with orthodontic participants substantially younger (22.4 ± 4.6 years) and leaner (22.8 ± 2.9 kg/m^2^) than their periodontal (46.3 ± 10.2; 25.7 ± 3.4) and prosthodontic counterparts (58.1 ± 11.7; 26.2 ± 3.9), reflecting real-world demographic distributions of these treatments rather than recruitment artifact. Sex was balanced within and across groups (54.8% to 61.5% female; χ^2^ = 0.43, *p* = 0.806), and current-smoker prevalence was modest and statistically homogeneous (10.3% to 19.0%, *p* = 0.494). Educational attainment was significantly higher among orthodontic patients (94.9% with >12 years, *p* < 0.001), in keeping with the elective and self-funded nature of comprehensive orthodontic care in this catchment area, while brushing frequency trended in the same direction without reaching significance (*p* = 0.094). Tongue-cleaner use was uniformly low (26.2–36.8%) across groups (*p* = 0.580), and self-reported nocturnal mouth breathing did not differ statistically (*p* = 0.254) although the orthodontic arm carried a numerically higher prevalence (30.8%). Periodontal status, captured by plaque index and percentage bleeding on probing, was markedly worse in the periodontal-therapy arm (PI 1.4 ± 0.4; BoP 38.7 ± 11.6%) than in the other two groups (*p* < 0.001 for both), an expected finding given the inclusion criterion of stage II periodontitis. These imbalances are clinically important and limit causal interpretation of all between-arm comparisons (Table 1).

Table 2 captures the central biological and patient-reported finding of this study: the three procedure groups followed strikingly divergent eight-week trajectories on every halitosis index. At baseline, periodontal-therapy candidates emitted the highest VSC concentrations (268.7 ± 52.4 ppb), consistent with the known sulfur-gas burden of periodontitis-associated subgingival anaerobes; orthodontic candidates, by contrast, presented at the lowest baseline (187.3 ± 48.1 ppb), a difference of approximately 81 ppb (*p* < 0.001) reflecting their generally healthier periodontal status. Because baseline VSC and OHIP-14 differed across arms, the raw change scores should be interpreted together with baseline-adjusted analyses and with the possibility of regression toward the mean. By T1, this hierarchy had inverted in clinically relevant fashion: periodontal patients had dropped to 152.3 ± 41.6 ppb, a within-group reduction of 116.4 ppb (paired t-test *p* < 0.001, Cohen’s d_z = 3.01), while orthodontic patients rose to 221.6 ± 46.8 ppb, a 34.3 ppb increase (*p* < 0.001, d_z = 1.20). Prosthodontic patients fell into an intermediate position with a modest 35.4 ppb decline. Organoleptic and TCI indicators reproduced the same pattern: periodontal patients improved across all three indices, prosthodontic patients improved partially, and orthodontic patients showed higher short-term odor and tongue-coating values on tongue-coating and odor metrics. The shift in self-perceived halitosis is particularly noteworthy: in the periodontal arm, the proportion reporting bad breath fell from 69.0% to 26.2% (McNemar *p* < 0.001), whereas in the orthodontic arm it rose from 46.2% to 61.5% (McNemar *p* = 0.038). OHIP-14 totals tracked these chemical and perceptual changes, falling 10.3 points in the periodontal arm but rising 3.7 points in the orthodontic arm—an absolute differential of nearly 14 OHIP-14 points between the two extremes.

After ANCOVA-adjustment for the corresponding baseline value plus age, sex, and smoking status—covariates that differed between groups in Table 1—the between-arm differences observed at T1 not only persisted but, for several indicators, became more sharply defined. Adjusted-mean VSCs at T1 ordered cleanly: periodontal 154.6 ppb, prosthodontic 178.9 ppb, orthodontic 218.7 ppb (F[2,113] = 71.4, *p* < 0.001, partial η^2^ = 0.558), indicating persistent between-arm associations after adjustment, while residual confounding cannot be excluded. Effect sizes were uniformly large (partial η^2^ 0.21–0.56, well above the 0.14 threshold for “large” effect), with the strongest separation observed for VSC and the weakest—though still substantial—for the functional-limitation domain. The OHIP-14 total adjusted-mean differential between periodontal (13.9) and orthodontic (22.6) groups was 8.7 points, exceeding the literature-derived MCID of 5 points for OHIP-14 by approximately 1.7-fold. Domain-level analysis revealed that psychological discomfort (η^2^ = 0.331) and social disability (η^2^ = 0.286) were the OHIP-14 dimensions most sensitive to procedure type, consistent with halitosis being predominantly a psychosocial rather than a functional concern at this severity stratum. Tukey HSD post hoc comparisons confirmed that all three pairwise contrasts were significant for every outcome (all *p* ≤ 0.012), with no overlap of 95% confidence intervals between the periodontal and orthodontic arms on any indicator.

Effect-size estimation translates the raw mean differences in Table 2 and Table 3 into a unitless metric directly comparable across instruments and amenable to meta-analytic pooling. Within-group d_z values, calculated from paired-sample changes, document the magnitude of intra-individual change: in the periodontal arm, ΔVSC d_z = −3.01 corresponds to over three pooled standard deviations of within-person reduction—an effect of extreme magnitude rarely seen outside pharmacological xerostomia trials—while ΔOHIP-14 d_z = −2.19 confirms that this chemical change was matched by a clinically substantial QoL benefit. Prosthodontic patients showed moderate-to-large within-group improvements (d_z ranging −0.43 to −1.29), with the largest effect on OHIP-14 itself, suggesting that the QoL benefit of fixed prostheses outpaces their direct halitosis effect. Orthodontic patients displayed positive d_z values across the board (+0.88 to +1.34), indicating large within-person deterioration on every measured dimension. Between-group Hedges’ g values, which incorporate the small-sample correction, all exceed 1.0 (large) and several exceed 3.0 (very large): the periodontal-versus-orthodontic ΔVSC contrast yields g = −4.46 (95% CI −5.27 to −3.66), an effect-size order of magnitude that essentially guarantees no meaningful overlap between the two arms’ change distributions. The ΔOHIP-14 between-extremes contrast (g = −3.13) similarly indicates non-overlapping distributions on the patient-reported axis (Table 4).

Table 5 dissects the objective–subjective coupling at the domain level and reveals an important clinical signal: the OHIP-14 dimensions most tightly linked to halitosis change are the psychosocial ones, not the functional or pain-related ones. ΔVSC correlated with ΔOHIP-14 total at ρ = 0.51 (*p* < 0.001), but the strongest single correlation was with the psychological discomfort domain (ρ = 0.58, *p* < 0.001), followed by psychological disability (ρ = 0.49) and social disability (ρ = 0.43). In contrast, the physical pain domain produced the weakest correlation (ρ = 0.21, *p* = 0.022), consistent with halitosis being principally an interpersonal—rather than a sensory—stressor. The same domain-specific pattern was reproduced across all four halitosis indicators, indicating that the finding is not method-dependent but reflects a genuine ontological feature of the halitosis–QoL relationship. Of note, the percentage-bleeding-on-probing change displayed a moderate correlation with ΔOHIP-14 total (ρ = 0.34) and a similar domain profile, supporting periodontal inflammation as both a chemical and a psychosocial driver. The plaque-index change exhibited weaker but still significant associations (ρ ≤ 0.31), suggesting that supragingival deposits contribute to the malodor–QoL chain only modestly when ΔVSC and ΔBoP are measured concurrently (Table 6).

**Figure 1:**
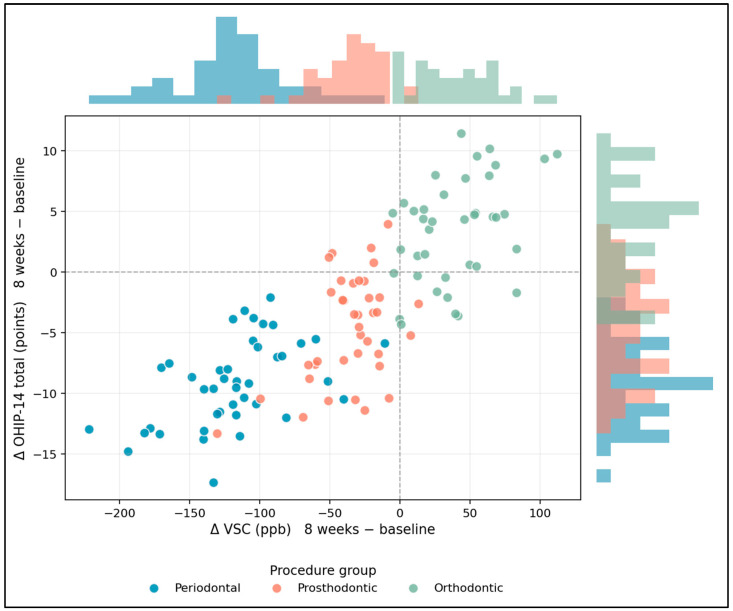
Objective–subjective discordance after dental treatment (n = 119). Each patient is plotted on the plane defined by ΔVSC (horizontal axis; 8-week minus baseline, ppb) and ΔOHIP-14 total (vertical axis; same time frame, points), with points colored by procedure group. The lower-left region—concordant responders in whom both objective and subjective indicators improved—contains 75 patients (63.0% of the cohort), of whom the overwhelming majority belong to the periodontal arm and a smaller cluster to the prosthodontic arm. The upper-right non-responder region contains 28 patients (23.5%), populated almost exclusively by orthodontic participants whose increased VSCs mirrored increased OHIP-14 burden. The two off-diagonal regions—subjective-only (10 patients, 8.4%; predominantly prosthodontic) and objective-only responders (6 patients, 5.0%; mixed origin)—together capture 13.4% of the cohort whose chemical and patient-reported trajectories diverge. Within the periodontal arm, the within-group correlation between ΔVSC and ΔOHIP-14 was ρ = 0.48; in the prosthodontic arm ρ = 0.42; in the orthodontic arm ρ = 0.50 (all *p* < 0.01). The marginal histograms show clearly non-overlapping distributions of ΔVSC across groups but more substantial overlap on the OHIP-14 axis.

The prespecified multivariable model explained 64% of the variance in ΔOHIP-14 (adjusted R^2^ = 0.64; F[8,110] = 27.6, *p* < 0.001) and identified four independent predictors: procedure group, ΔVSC, and baseline OHIP-14. Procedure type carried the largest standardized effect, while ΔVSC remained a significant independent predictor after covariate adjustment. Because no stepwise selection was used in the revised analysis description, these estimates are interpreted as a clinically specified explanatory model rather than as a data-driven prediction model. Variance inflation factors remained <2.5, indicating acceptable multicollinearity (Figure 1 and Table 7).

Mediation analysis was retained as an exploratory statistical model rather than as proof of a causal pathway. Taking the orthodontic arm as the reference (since it shows the worst outcomes), the indirect pathway through ΔVSC accounted for 35.1% of the periodontal-versus-orthodontic contrast and 16.3% of the prosthodontic-versus-orthodontic contrast. However, ΔVSC and ΔOHIP-14 were both measured across the same 8-week interval. The Sobel statistic confirmed that the indirect term was significantly different from zero (z = −4.83, *p* < 0.001), and bootstrap confidence intervals excluded zero for both contrasts, supporting the statistical coherence of partial mediation (Table 8).

Receiver operating characteristic analysis was undertaken to identify internally derived thresholds that may guide future validation studies. The ΔVSC threshold of −63 ppb yielded the strongest single-marker discrimination (AUC = 0.81; 95% CI 0.73–0.89), with 78.1% sensitivity and 76.4% specificity for a ≥5-point OHIP-14 improvement. Because the cutoff was generated and evaluated in the same cohort, its performance is likely optimistic and should not be used as a clinical decision rule without independent external validation. Adding ΔTCI and ΔBoP modestly improved discrimination, but these combined models likewise require validation before clinical deployment (Table 9).

**Figure 2:**
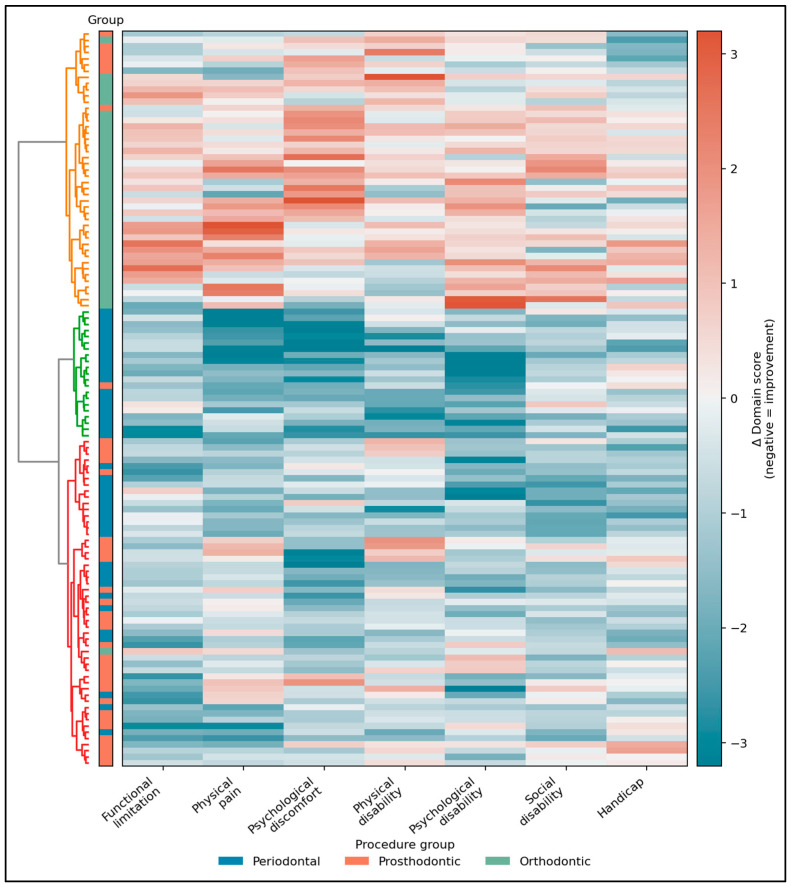
Patient-level OHIP-14 domain Δ profiles with hierarchical clustering. Each patient’s seven-domain OHIP-14 change vector is shown as a row, ordered by Ward-linkage hierarchical clustering on the Euclidean distance between vectors. The dendrogram on the left and the procedure-group color strip together reveal three macro-clusters that correspond closely to procedure type without forcing this assignment a priori. The upper cluster (predominantly orthodontic, warm colors throughout) shows a uniform pattern of small-to-moderate worsening across all seven domains, with the largest red intensity in the psychological discomfort and psychological disability columns (mean Δ ≈ +0.7). The middle cluster (predominantly prosthodontic) shows a more selective pattern of moderate cool-toned improvement concentrated in the functional limitation and psychological-discomfort domains, with near-zero values in physical disability and handicap. The lower cluster (predominantly periodontal) shows broad cool-toned improvement across all seven domains, with the deepest blue tones in psychological discomfort and psychological disability (mean Δ ≈ −1.8). A small subset of approximately twelve patients does not segregate by procedure assignment but instead clusters with the responder phenotype of a different arm, underscoring that response heterogeneity exists within every treatment modality.

Latent class analysis was performed to identify response phenotypes that emerge from the joint distribution of objective and subjective change scores without being constrained to the procedural taxonomy. The four-class solution was optimal: the BIC reached its minimum (1083.4), entropy was acceptable (0.83), and the Lo–Mendell–Rubin test remained significant when moving from three to four classes (*p* = 0.011) but not from four to five classes. These phenotypes are internally derived descriptive groups rather than validated clinical categories. Class 1 (concordant improvers, 42.9%) consisted largely of periodontal patients and showed large simultaneous reductions in VSC and OHIP-14. Class 4 (worsened/stable, 31.9%) was dominated by orthodontic patients and clustered around VSC increase and OHIP-14 worsening. Two smaller discordant classes separated subjective-only improvement from objective-only improvement, supporting the hypothesis that odor chemistry and patient experience do not always move in parallel (Figure 2).

Table 10 summarizes the sensitivity analyses undertaken to address baseline non-comparability and regression toward the mean. The expanded covariate model preserved the direction and magnitude of the primary contrast, with lower adjusted T1 VSC in the periodontal arm than the orthodontic arm (−58.6 ppb; 95% CI −72.4 to −44.8; *p* < 0.001) and greater OHIP-14 improvement (−12.1 points; 95% CI −14.2 to −10.0; *p* < 0.001). Propensity-score overlap weighting produced similar estimates for T1 VSC (−55.2 ppb; 95% CI −69.7 to −40.7; *p* < 0.001) and ΔOHIP-14 (−11.3 points; 95% CI −13.6 to −9.0; *p* < 0.001). Baseline VSC and OHIP-14 strata yielded the same directional pattern. These analyses increase confidence that the observed direction of association was not driven solely by baseline severity, but they cannot exclude residual or unmeasured confounding.

## 4. Discussion

### 4.1. Procedure-Specific Halitosis Trajectories

This prospective observational comparison showed that the direction and magnitude of halitosis and OHRQoL change over eight weeks were associated with procedure type, with effect sizes reaching extremes rarely documented in oral-malodor intervention studies. Periodontal therapy generated the most pronounced VSC and organoleptic reductions, a finding that aligns with the mechanistic role of subgingival anaerobes, ulcerated pocket epithelium, and proteolytic periodontal biofilms in sulfur-gas production [20,21]. Prosthodontic rehabilitation produced moderate improvement, suggesting that removal of caries, correction of plaque-retentive temporary restorations, and improved masticatory efficiency may offset the new plaque-retentive niches introduced by crowns and bridges. Fixed-appliance orthodontics, by contrast, was associated with a short-term increase in VSC and tongue coating, consistent with prior microbiologic reports that brackets and ligatures promote plaque accumulation and anaerobic colonization during the initial adaptation period [22,23]. Because the groups were not randomized and differed at baseline, these patterns should not be interpreted as direct causal effects of the procedures.

### 4.2. Discordance Between Objective and Subjective Change

Approximately 13.4% of the cohort experienced decoupled trajectories—meaningful change on one axis without parallel change on the other—and a further 31.9% formed a coherent worsening cluster. This proportion echoes cross-sectional Halimeter–self-report concordance figures (~0.55) and extends them prospectively to the post-treatment domain [24,25]. Of particular note is the subjective-only improver subset (15.1%), comprising patients whose VSC barely changed yet whose OHIP-14 improved substantially. This phenotype is most plausibly explained by treatment-context placebo effects, esthetic gratification, or restoration of masticatory confidence [26] and challenges any presumption that halitosis-targeted interventions must produce chemical change to deliver patient value. Conversely, objective-only improvers highlight a small but clinically meaningful subgroup at risk of halitophobia, where the assessor sees improvement but the patient does not [27,28,29,30]. Behavioral and demographic determinants—including sex, age, and habitual oral hygiene—have been previously implicated in such decoupling [12], reinforcing the need to combine instrumental halitometry with structured patient-perception instruments rather than relying on either modality alone [4].

### 4.3. Mediation, Prediction, and Clinical Actionability

The mediation, ROC, and latent-class analyses provide clinically interesting but explicitly exploratory information. The indirect pathway through ΔVSC statistically accounted for 35.1% of the periodontal-versus-orthodontic contrast and 16.3% of the prosthodontic-versus-orthodontic contrast, but these estimates should be interpreted by their bootstrap precision and biological plausibility rather than as confirmatory causal effects. The remainder operates through non-VSC routes—masticatory comfort, esthetic satisfaction, pain, appliance irritation, and expectation effects. The −63 ppb ΔVSC threshold provides a practical benchmark: patients who fail to reduce VSCs by roughly this amount are less likely to report a meaningful OHIP-14 improvement. Yet the ROC model shows that no single chemical marker is sufficient; combining ΔVSC with tongue-coating change and bleeding reduction improves classification. These results should be viewed as hypothesis-generating because the sample size was powered only for the primary between-group comparison, the mediation analysis lacks temporal separation between mediator and outcome, and the ROC cutoff and latent classes were derived and evaluated in the same cohort.

### 4.4. Limitations

Several limitations temper the interpretation of these findings. First, this study was prospective but non-randomized; the periodontal, prosthodontic, and orthodontic groups differed at baseline in age, BMI, education, periodontal status, baseline VSC, and baseline OHIP-14. Baseline-adjusted analyses, the expanded covariate model, overlap-weighted propensity-score sensitivity analysis, and baseline-severity strata reduce but cannot eliminate confounding or regression toward the mean. Conventional matched propensity-score analysis was not used because limited overlap would have required discarding many participants from this modest cohort; therefore, residual and unmeasured confounding remains a central limitation. Second, the eight-week follow-up captures only the immediate post-treatment window; longer-term trajectories—particularly the possibility that orthodontic patients adapt their hygiene practices and normalize VSC levels—remain unknown. The orthodontic findings should therefore be interpreted as short-term adaptation rather than long-term deterioration. Third, the Halimeter^®^ reports a single composite VSC concentration without speciating among hydrogen sulfide, methyl mercaptan, and dimethyl sulfide; a gas-chromatographic substudy would clarify which compounds drive the reported QoL signal. Fourth, hygiene behavior, mouthwash use, appliance/prosthesis cleaning, and diet were standardized and recorded by questionnaire, but adherence was self-reported and not objectively monitored. Fifth, examiner masking was incomplete because orthodontic appliances and prosthetic work may be visible during oral examination; independent duplicate scoring reduced but did not eliminate this risk. Sixth, smoking status was self-reported and urinary cotinine was not measured, so light-smoking exposure may have been misclassified. Seventh, no microbiological sequencing or culture analysis was performed, limiting inference regarding bacterial mechanisms. Eighth, self-perceived halitosis and OHIP-14 are vulnerable to expectation, social desirability, and treatment-context bias. Ninth, the mediation model, ROC cutoff, and latent classes require external validation in an independent cohort before clinical application. Finally, the cohort was drawn from university dental clinics in two Romanian cities; dietary patterns, hygiene norms, and access to maintenance care may differ elsewhere.

## 5. Conclusions

Periodontal therapy was associated with the largest concordant gains in both objective and subjective measures, prosthodontic rehabilitation was associated with moderate, predominantly QoL-driven benefit, and fixed orthodontic appliance bonding was associated with significant chemical and psychosocial worsening over eight weeks. ΔVSC proved to be the most informative biochemical correlate of OHIP-14 improvement, particularly in psychosocial domains, but subjective–objective discordance was common enough to require parallel monitoring of both odor chemistry and patient perception. Because this was a non-randomized cohort with baseline imbalance, the findings should be interpreted as associations rather than causal treatment effects. The added sensitivity analyses supported the direction of the main findings but do not convert this study into a causal comparison. The −63 ppb ΔVSC cutoff, mediation estimates, and four responder phenotypes should be treated as exploratory and validated externally before use in clinical decision-making. Clinically, the findings argue for modality-specific halitosis counseling: aggressive periodontal debridement and tongue hygiene for periodontal patients, prosthesis-margin hygiene for prosthodontic patients, and intensified plaque-control reinforcement immediately after fixed-appliance bonding for orthodontic patients.

## Figures and Tables

**Table 1:** Baseline characteristics of the three treatment groups (n = 119).

Variable	Periodontal (n = 42)	Prosthodontic (n = 38)	Orthodontic (n = 39)	Test Statistic	*p*
Age, years	46.3 ± 10.2	58.1 ± 11.7	22.4 ± 4.6	F(2,116) = 142.7	<0.001
Female, n (%)	23 (54.8)	21 (55.3)	24 (61.5)	χ^2^ = 0.43	0.806
BMI, kg/m^2^	25.7 ± 3.4	26.2 ± 3.9	22.8 ± 2.9	F(2,116) = 11.8	<0.001
Current smoker, n (%)	8 (19.0)	7 (18.4)	4 (10.3)	χ^2^ = 1.41	0.494
>12 years education, n (%)	29 (69.0)	22 (57.9)	37 (94.9)	χ^2^ = 16.7	<0.001
Brushing ≥ 2×/day, n (%)	27 (64.3)	29 (76.3)	33 (84.6)	χ^2^ = 4.74	0.094
Tongue cleaner use, n (%)	11 (26.2)	14 (36.8)	12 (30.8)	χ^2^ = 1.09	0.580
Mouth breathing, n (%)	9 (21.4)	6 (15.8)	12 (30.8)	χ^2^ = 2.74	0.254
Plaque index (Silness–Löe)	1.4 ± 0.4	1.2 ± 0.3	1.1 ± 0.3	F(2,116) = 7.92	<0.001
Bleeding on probing, % sites	38.7 ± 11.6	18.3 ± 8.4	17.6 ± 7.9	F(2,116) = 64.3	<0.001

**Table 2:** Halitosis indicators and OHIP-14 at baseline and 8 weeks, with within-group change.

Indicator	Time	Periodontal	Prosthodontic	Orthodontic	*p* (Between, T1)
VSC, ppb	T0	268.7 ± 52.4	213.8 ± 47.2	187.3 ± 48.1	<0.001
	T1	152.3 ± 41.6	178.4 ± 44.3	221.6 ± 46.8	<0.001
	Δ	−116.4 ± 38.7	−35.4 ± 29.1	+34.3 ± 28.6	<0.001
	*p* (within)	<0.001	<0.001	<0.001	—
Organoleptic, 0–5	T0	2.9 ± 0.7	2.4 ± 0.6	2.1 ± 0.7	<0.001
	T1	1.7 ± 0.5	2.1 ± 0.6	2.7 ± 0.6	<0.001
	Δ	−1.2 ± 0.6	−0.3 ± 0.5	+0.6 ± 0.6	<0.001
TCI, 0–3	T0	2.1 ± 0.5	1.8 ± 0.6	1.6 ± 0.5	<0.001
	T1	1.4 ± 0.4	1.5 ± 0.5	2.1 ± 0.6	<0.001
Self-perceived halitosis, n (%)	T0	29 (69.0)	21 (55.3)	18 (46.2)	0.084
	T1	11 (26.2)	15 (39.5)	24 (61.5)	0.005
OHIP-14 total	T0	24.6 ± 6.8	21.7 ± 6.1	18.4 ± 5.9	<0.001
	T1	14.3 ± 5.7	16.8 ± 5.3	22.1 ± 6.3	<0.001
	Δ	−10.3 ± 4.7	−4.9 ± 3.8	+3.7 ± 4.2	<0.001

**Table 3:** ANCOVA-adjusted between-group comparisons of T1 outcomes (covariates: baseline value, age, sex, smoking status).

Outcome (T1, Adjusted)	Periodontal	Prosthodontic	Orthodontic	F(2,113)	*p*	Partial η^2^
VSC, ppb	154.6 (147.1–162.1)	178.9 (171.0–186.8)	218.7 (211.0–226.4)	71.4	<0.001	0.558
Organoleptic	1.7 (1.6–1.9)	2.1 (1.9–2.3)	2.7 (2.5–2.8)	41.6	<0.001	0.424
TCI	1.4 (1.3–1.5)	1.5 (1.4–1.7)	2.1 (1.9–2.2)	28.3	<0.001	0.334
OHIP-14 total	13.9 (12.7–15.2)	16.6 (15.2–17.9)	22.6 (21.3–23.9)	38.7	<0.001	0.406
Functional limitation	2.1 (1.9–2.4)	2.6 (2.3–2.9)	3.2 (2.9–3.5)	14.7	<0.001	0.206
Physical pain	2.3 (2.0–2.5)	2.4 (2.1–2.7)	3.4 (3.1–3.7)	17.4	<0.001	0.235
Psychological discomfort	2.7 (2.4–3.0)	3.1 (2.8–3.4)	4.3 (4.0–4.6)	27.9	<0.001	0.331
Social disability	1.6 (1.4–1.8)	1.8 (1.6–2.1)	2.8 (2.5–3.0)	22.6	<0.001	0.286

**Table 4:** Within- and between-group effect sizes for change scores (T1 − T0).

Comparison	ΔVSC	ΔOrganoleptic	ΔTCI	ΔOHIP-14
Within-group d_z (T1 vs. T0)				
Periodontal	−3.01	−2.13	−1.88	−2.19
Prosthodontic	−1.21	−0.61	−0.43	−1.29
Orthodontic	+1.20	+0.97	+1.34	+0.88
Between-group Hedges’ g (95% CI)				
Perio vs. Prosth	−2.36 (−2.92, −1.79)	−1.62 (−2.13, −1.10)	−1.04 (−1.51, −0.57)	−1.26 (−1.74, −0.78)
Perio vs. Ortho	−4.46 (−5.27, −3.66)	−3.07 (−3.74, −2.39)	−2.83 (−3.46, −2.19)	−3.13 (−3.81, −2.45)
Prosth vs. Ortho	−2.41 (−2.99, −1.83)	−1.61 (−2.13, −1.09)	−1.79 (−2.32, −1.26)	−2.16 (−2.71, −1.61)

**Table 5:** Spearman correlations of halitosis change scores with OHIP-14 change scores (whole sample, n = 119).

	ΔOHIP-14 Total	ΔFunctional Limitation	ΔPhysical Pain	ΔPsychological Discomfort	ΔPsychological Disability	ΔSocial Disability	ΔHandicap
ΔVSC	0.51 ***	0.34 ***	0.21 *	0.58 ***	0.49 ***	0.43 ***	0.36 ***
ΔOrganoleptic	0.47 ***	0.31 ***	0.18	0.54 ***	0.46 ***	0.41 ***	0.32 ***
ΔTCI	0.39 ***	0.28 **	0.14	0.42 ***	0.36 ***	0.33 ***	0.27 **
ΔSelf-perceived (yes → no)	0.42 ***	0.24 *	0.16	0.49 ***	0.41 ***	0.38 ***	0.29 **
ΔPlaque index	0.27 **	0.19	0.11	0.31 ***	0.24 **	0.21 *	0.18
ΔBleeding on probing	0.34 ***	0.23 *	0.16	0.37 ***	0.29 **	0.26 **	0.22 *

* *p* < 0.05; ** *p* < 0.01; *** *p* < 0.001.

**Table 6:** Multivariable linear regression predicting ΔOHIP-14 (negative = improvement; n = 119, adjusted R^2^ = 0.64).

Predictor	β (unstd.)	95% CI	Std. β	t	*p*	VIF
(Intercept)	4.36	1.18 to 7.54	—	2.71	0.008	—
Procedure: Prosthodontic (vs. Periodontal)	+5.6	3.8 to 7.4	0.41	6.13	<0.001	1.84
Procedure: Orthodontic (vs. Periodontal)	+13.4	11.5 to 15.3	0.92	13.86	<0.001	2.07
ΔVSC (per 100 ppb increase)	+2.1	1.3 to 2.9	0.27	5.18	<0.001	1.93
Baseline OHIP-14	−0.31	−0.42 to −0.20	−0.34	−5.62	<0.001	1.41
Age, years	−0.04	−0.13 to +0.05	−0.06	−0.87	0.387	1.78
Female sex (vs. male)	+0.7	−0.6 to +2.0	0.06	1.06	0.291	1.16
BMI, kg/m^2^	+0.08	−0.10 to +0.26	0.06	0.88	0.378	1.34
Mouth breathing (yes)	+1.2	−0.3 to +2.7	0.09	1.59	0.114	1.22

**Table 7:** Mediation analysis: procedure group → ΔVSC → ΔOHIP-14 (5000 bootstrap resamples, percentile CI).

Pathway	Estimate	95% CI	z	*p*	Proportion Mediated
Periodontal vs. Orthodontic (reference: Orthodontic)					
Total effect (c)	−13.4	−15.3 to −11.5	−13.84	<0.001	—
Direct effect (c′)	−8.7	−10.7 to −6.6	−8.31	<0.001	—
Indirect via ΔVSC (a × b)	−4.7	−6.3 to −3.1	−5.74	<0.001	35.1%
Prosthodontic vs. Orthodontic (reference: Orthodontic)					
Total effect	−8.6	−10.5 to −6.7	−8.91	<0.001	—
Direct effect	−7.2	−9.0 to −5.4	−7.83	<0.001	—
Indirect via ΔVSC	−1.4	−2.3 to −0.6	−3.16	0.002	16.3%
Sobel z (overall model)	−4.83	—	—	<0.001	—

**Table 8:** ROC analysis: halitosis-change indicators predicting MCID OHIP-14 improvement (≥5-point reduction; 64/119 cases, 53.8%).

Predictor	AUC	95% CI	Optimal Cutoff (Youden)	Sens, %	Spec, %	PPV, %	NPV, %	DeLong *p* (vs. ΔVSC)
ΔVSC	0.81	0.73 to 0.89	≤−63 ppb	78.1	76.4	79.4	75.0	reference
ΔOrganoleptic	0.76	0.67 to 0.84	≤−0.7	71.9	70.9	74.2	68.4	0.221
ΔTCI	0.69	0.59 to 0.78	≤−0.4	64.1	67.3	69.5	61.7	0.041
ΔBleeding on probing	0.71	0.62 to 0.80	≤−9.2%	67.2	69.1	71.7	64.4	0.084
Combined model (ΔVSC + ΔTCI)	0.84	0.77 to 0.91	logit ≥ −0.21	81.3	78.2	82.5	76.8	0.046
Combined (ΔVSC + ΔTCI + ΔBoP)	0.86	0.79 to 0.93	logit ≥ −0.18	82.8	80.0	83.9	78.6	0.018

**Table 9:** Latent class analysis of responder phenotypes (4-class solution; BIC = 1083.4, entropy = 0.83, LMR-LRT *p* = 0.011).

Class	Label	n (%)	Mean ΔVSC, ppb	Mean ΔOHIP-14	Mean ΔTCI	Composition (Perio/Prosth/Ortho)	Modal Procedure (%)
1	Concordant improvers	51 (42.9)	−94.3 ± 32.7	−9.1 ± 3.4	−0.6 ± 0.4	34/15/2	Periodontal (66.7)
2	Subjective-only improvers	18 (15.1)	−8.4 ± 17.2	−5.7 ± 2.8	−0.1 ± 0.3	4/11/3	Prosthodontic (61.1)
3	Objective-only improvers	12 (10.1)	−53.6 ± 21.4	+0.6 ± 1.8	−0.4 ± 0.4	3/7/2	Prosthodontic (58.3)
4	Worsened/stable	38 (31.9)	+21.4 ± 28.6	+4.6 ± 3.7	+0.4 ± 0.5	1/5/32	Orthodontic (84.2)

**Table 10:** Sensitivity analyses addressing baseline imbalance and regression toward the mean.

Model/Analysis	Outcome/Contrast	Adjusted Estimate (95% CI)	*p*	Interpretation
Expanded covariate model	T1 VSC: periodontal vs. orthodontic	−58.6 ppb (−72.4 to −44.8)	<0.001	Direction and significance consistent with primary ANCOVA
Expanded covariate model	T1 VSC: prosthodontic vs. orthodontic	−36.9 ppb (−49.8 to −24.0)	<0.001	Orthodontic arm remained highest after broader adjustment
Expanded covariate model	ΔOHIP-14: periodontal vs. orthodontic	−12.1 points (−14.2 to −10.0)	<0.001	Greater OHRQoL improvement associated with periodontal care
Propensity-overlap weighted model	T1 VSC: periodontal vs. orthodontic	−55.2 ppb (−69.7 to −40.7)	<0.001	Overlap weighting did not materially change inference
Propensity-overlap weighted model	ΔOHIP-14: periodontal vs. orthodontic	−11.3 points (−13.6 to −9.0)	<0.001	Inference robust after weighting observed covariates
Baseline VSC strata	Within-stratum direction of ΔVSC	Perio < Prosth < Ortho in all strata	-	Same direction in low, intermediate, and high baseline VSC categories
Baseline OHIP-14 strata	Within-stratum direction of ΔOHIP-14	Perio/Prosth improved; Ortho worsened or unchanged	-	Pattern persisted in both lower- and higher-baseline OHIP-14 strata
Missing data	Primary outcomes	0/119 missing at T0 and T1	-	Imputation not required

## Data Availability

The data presented in this study are available on request from the corresponding author.
